# Case report: immunotherapy successfully treated brain metastasis in intrahepatic cholangiocarcinoma and literature review

**DOI:** 10.3389/fonc.2022.911202

**Published:** 2022-08-03

**Authors:** Peiyi Xie, Lei Guo, Bo Zhang, Yongfeng Xu, Qi Song, Hongcheng Shi, Qinghai Ye, Hui Li, Yongsheng Xiao

**Affiliations:** ^1^ Department of Liver Surgery and Transplantation, Key Laboratory of Carcinogenesis and Cancer Invasion, Ministry of Education, Liver Cancer Institute, Zhongshan Hospital, Fudan University, Shanghai, China; ^2^ Department of Pathology, Zhongshan Hospital, Fudan University, Shanghai, China; ^3^ Department of Nuclear Medicine, Zhongshan Hospital, Fudan University, Shanghai, China; ^4^ Shanghai Medical College and Zhongshan Hospital Immunotherapy Technology Transfer Center, Fudan University, Shanghai, China

**Keywords:** brain metastasis, advanced intrahepatic cholangiocarcinoma (iCCA), immune checkpoint inhibitors (ICIs), PD1, immunotherapy

## Abstract

Brain metastasis from intrahepatic cholangiocarcinoma (iCCA) is extremely rare, and no standard therapeutic strategy has been established. Camrelizumab is a programmed cell death protein 1 (PD-1) inhibitor that has been widely studied in treating liver cancer. Combined immunotherapy and targeted therapy are a promising approach for treating advanced iCCA. Despite that immune checkpoint inhibitor (ICI)-based neoadjuvant therapy on iCCA has shown a significant response rate and resection rate, few reports have shown the therapeutic efficacy of immunotherapy in treating brain metastasis from iCCA. Although PD-1 inhibitors such as pembrolizumab, nivolumab, or camrelizumab are increasingly applied in clinic practice to treat multiple malignancies, to the best of our knowledge, we report the first case of an iCCA patient with brain metastasis successfully treated with a combined immunotherapy and targeted therapy. The patient is a 54-year-old man with metastatic iCCA in brain treated though camrelizumab plus lenvatinib therapy with a complete response (CR). By the time of writing, he has had a progression-free survival of 17.5 months and did not experience any severe side effects related to this therapy. Camrelizumab plus lenvatinib therapy showed favorable efficacy and manageable toxicity for this patient with advanced iCCA and could be of interest for more prospective randomized trials to further verify the potential clinical benefits.

## Introduction

Cholangiocarcinoma (CCA) is the second most common primary liver cancer arising from the hepatic biliary system (1). It is a rare malignancy accounting for around 10%–20% of all hepatic malignancies and has poor prognosis as patients usually have metastatic diseases when being diagnosed (2). Regional lymph nodes and adjacent organs are the most common metastatic sites for CCA (3). However, CCA with brain metastasis is extremely rare and the prognosis is very poor (4). In treating these patients, no treatment guidelines exist to date and common therapeutic strategies include surgical excision, radiation, and chemotherapy (1).

Advanced CCA remains a difficult-to-treat disease, and therapy remains palliative. Although chemotherapy with gemcitabine and cisplatin is considered as the standard first-line treatment for unresectable patients with advanced biliary tract cancer, the median overall survival is less than 1 year (5). Targeted therapies such as using isocitrate dehydrogenase 1 or 2 *(IDH1 or 2*) inhibitors and fibroblast growth factor receptor (*FGFR*) 2 have also been rapidly translated into promising therapeutic strategies in treating advanced CCA (6, 7). As a multitargeted kinase inhibitor, lenvatinib (E7080) can target PDGFR-b, VEGFR1-3, FGFR1–4, KIT, and RET (8). The published phase II clinical trials include lenvatinib with sorafenib in hepatocellular carcinoma (NCT01761266) and lenvatinib as monotherapy for treating unresectable biliary cancer (NCT02579616).

Treating biliary tract cancer with immune checkpoint inhibitors (ICIs) is the largest area of ongoing research among all immunotherapy strategies. ICIs combined with other therapies including chemotherapy, additional immunotherapies, and targeted therapies have all been approaches to increase response rates and improve outcomes (9). A recent study of Lin et al. demonstrated that pembrolizumab combined with lenvatinib is potentially effective and tolerable as a systemic therapy for patients with refractory bile tract carcinoma (10). Camrelizumab is a humanized monoclonal antibody against PD-1 which can block the PD-1/PD-L1 interaction and thus suppress the immune evasion of malignant cancer (11). Phase I trials have demonstrated positive antitumor effects of camrelizumab among patients with advanced solid tumor (12, 13). Here, we report the first case of a patient with intrahepatic cholangiocarcinoma (iCCA) brain metastasis successfully treated by camrelizumab plus lenvatinib combination therapy.

## Case report

**Figure 1:**
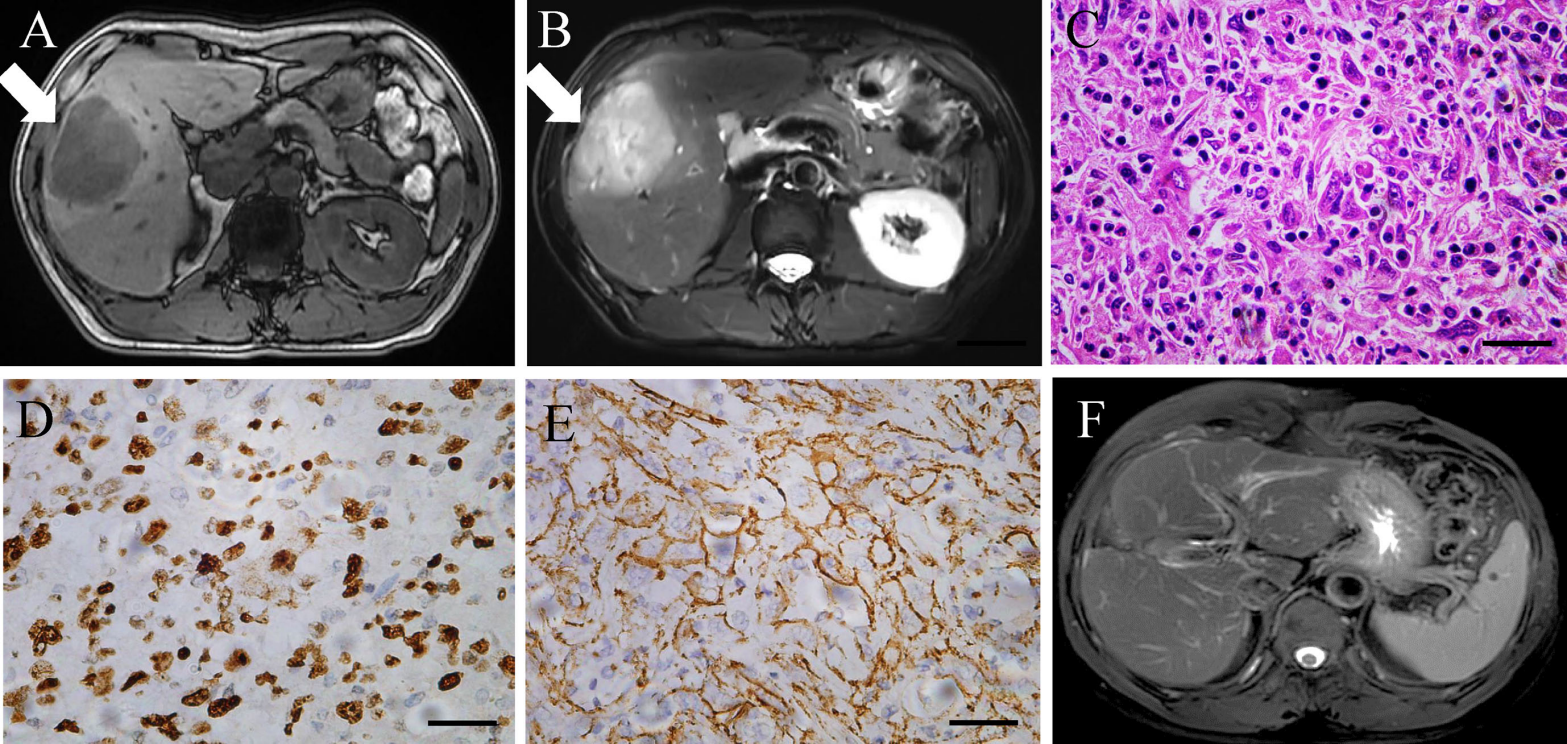
**(A, B)** Enhanced MRI scan of the liver lesion before surgery. **(C–E)** Histopathological finding (hematoxylin and eosin staining, Ki67, and β-catenin), poorly differentiated intrahepatic sarcomatoid cholangiocarcinoma. Magnification, ×400. Scale bar: 50 µm. **(F)** MRI scan of the liver lesion after surgery in February 2022.

A 54-year-old male patient with a history of chronic hepatitis B for more than 30 years was referred for a liver lesion detected by abdominal ultrasound because of abdominal pain. Enhanced abdominal magnetic resonance imaging (MRI) presented one tumor lesion (with a size of 6.5 × 6 × 5 cm) in segments V of liver (**Figures 1A**, **B**). Additionally, positron emission tomography-computed tomography (PET-CT) presented no extrahepatic metastasis of this patient. Laboratory tests showed that the tumor markers alpha-fetoprotein (AFP), carcinoembryonic antigen (CEA), and carbohydrate antigen (CA) 19.9 were negative and physical examination showed no remarkable findings. After a discussion with the medical team, our team performed special segmental hepatectomy combined with cholecystectomy and skeletal dissection of hepatoduodenal ligament together in July 2020. Tumor biopsies from resected liver lesions revealed a poorly differentiated intrahepatic sarcomatoid cholangiocarcinoma (ISSC), which is a rare histopathology classification of iCCA (**Figure 1C**). Moreover, histochemistry staining showed positivity for cytokeratin (CK), CK19, CK7, CD34, Ki67 (50% positive rate, **Figure 1D**), and β-catenin (**Figure 1E**) and negativity for AFP and hepatocyte specific antigen (Hep-Par-1). Chemotherapy with capecitabine was initiated in the second month after surgery, and no recurrence of the primary tumor had been found at the last follow-up in February 2022 (**Figure 1F**).

**Figure 2:**
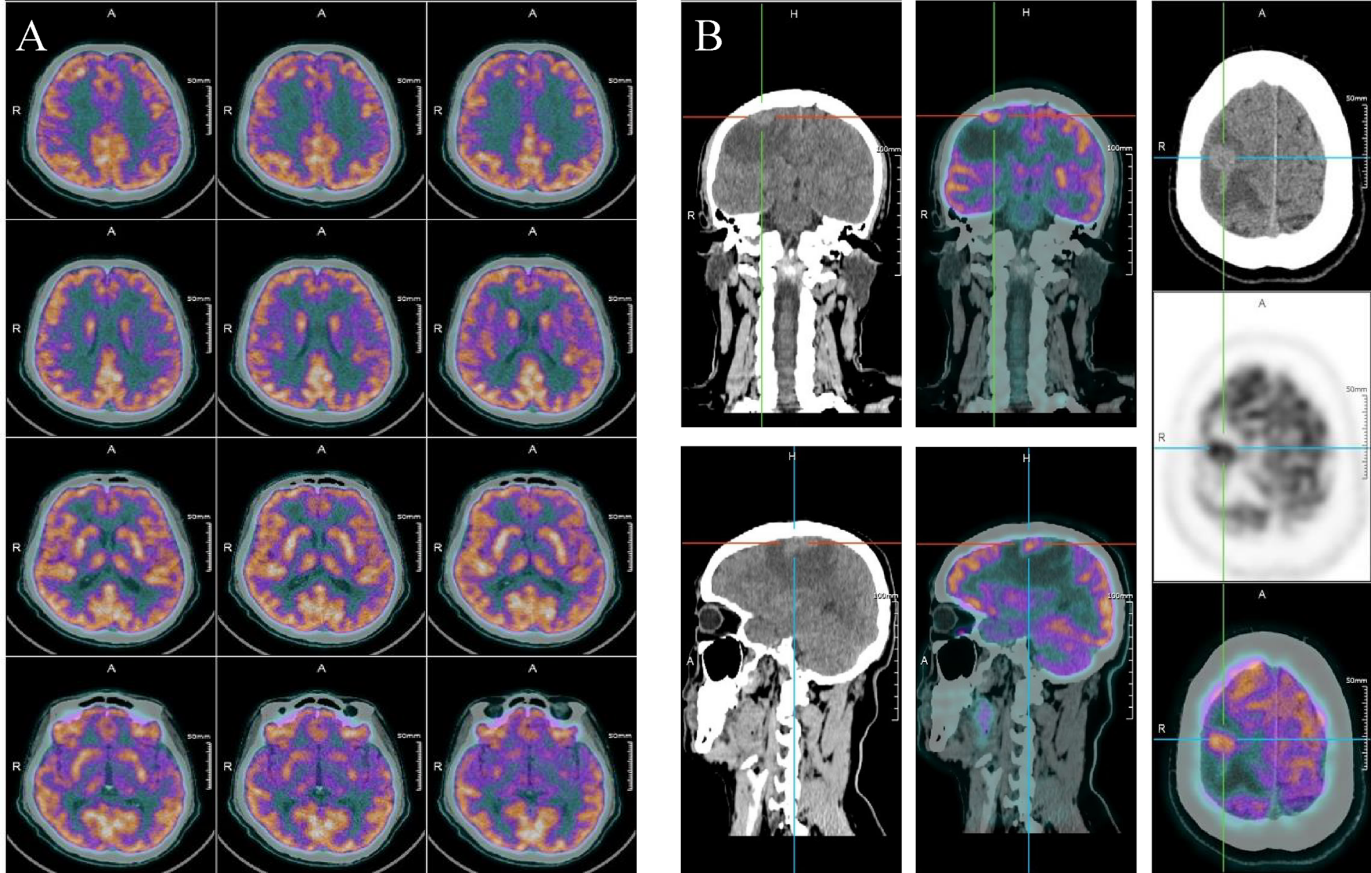
**(A)** PET-CT scan of the brain before liver surgery. **(B)** PET-CT scan of the brain 3 months after liver surgery.

Three months after surgery in October 2021, this patient complained weakness of left upper limb and left lower limb. Intracranial hypertension was diagnosed for this patient, and mannitol was applied to treat the increased intracranial pressure. In July 2021 before the liver surgery, no extrahepatic metastasis was found in the brain of this patient (**Figure 2A**). However, on this presentation, PET-CT revealed a 1.1 cm × 0.6 cm mass in his right frontal lobe (**Figure 2B**). As a result, our multidisciplinary team (MDT) diagnosed that the patient had brain metastasis of iCCA. According to the whole-exome sequencing (WES) performed on the resected tumor tissue, the tumor genomics of this patient exhibited mutations in KIT, TP53, and PDGFR. Considering no considerable evidence proving the effectiveness of operation and radiotherapy in treating patients with brain metastasis from iCCA, we decided to initiate the systemic treatment with camrelizumab (200 mg every 3 weeks) plus lenvatinib (8 mg orally once daily) therapy in October 2020.

**Figure 3:**
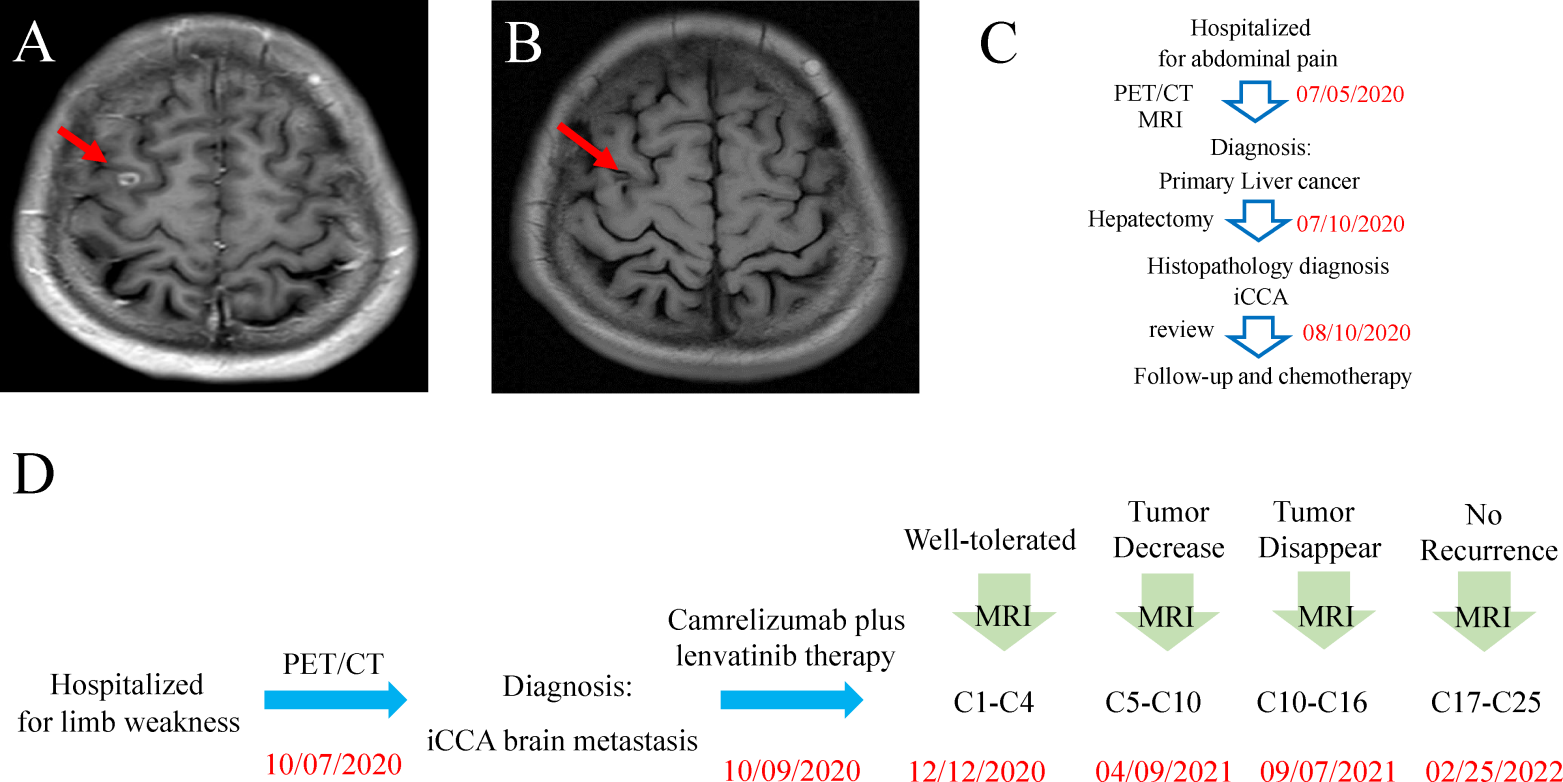
**(A)** Brain MRI scan after treatment with camrelizumab and lenvatinib for 4 cycles. **(B)** Brain MRI scan after treatment with camrelizumab and lenvatinib for 16 cycles. **(C)** Timeline of the disease course and treatment before brain metastasis. **(D)** Timeline of the disease course and treatment after brain metastasis.

After the first three-cycle treatment (approximately 2 months), the general condition of this patient remained stable, and the brain MRI presented a 0.8 × 0.7 mass in the right frontal lobe and little amount of surrounding edema (**Figure 3A**). In the following treatment cycles, this patient was followed up regularly. During this period, the previous metastatic lesions in the brain regressed gradually until complete regression was examined after 16-cycle treatments in September 2021 (**Figure 3B**). Additionally, abdominal and brain MRI presented no recurrence of the primary tumor and no occurrence of new focus. Overall, from June 2020 to February 2022, this patient admitted to our hospital twice. Although during the first admission, we successfully excised his liver tumor through surgery, the brain metastatic lesion was first found after 3 months. Fortunately, immunotherapy plus targeted therapy was effective and well-tolerated until the tumor lesion in the brain totally disappeared (**Figures 3C**, **D**).

By the time of writing, the patient had achieved a progression-free survival of 17.5 months since initiating the combination therapy. During this period, the patient remained a relatively stable physical state throughout the treatment and no evidence of active disease and clinically relevant adverse event was found.

## Discussion

In our article, we report a rare case with brain metastasis from iCCA and the metastatic lesion in the brain had a complete response to PD1 inhibitor camrelizumab plus lenvatinib combination therapy. After the 16-cycle PD1 inhibitor treatment, the metastatic lesion in the brain disappeared totally and the condition of this patient remained stable. Considering the low incidence of brain metastasis in iCCA patients, more of these patients are expected to be included in the future clinical trials to explore more effective and reliable therapeutic strategies. Thus, our report will supplement the existing literatures and provide valuable evidence for PD1 inhibitors in treating iCCA patients with brain metastasis.

Cholangiocarcinoma (CCA) consists of various malignancies occurring in the biliary tree. iCCA is a subtype of CCA and accounts for around 10%–20% of all CCAs (14). The prognosis for patients with iCCA is very poor, and the average 5-year overall survival (OS) rate is between 5% and 10% for patients with advanced iCCA (15). One of the most important reasons for causing the poor prognosis is the limited efficacy of traditional systemic and locoregional treatments such as chemotherapy and radiotherapy (16). To treat patients with advanced iCCA, gemcitabine plus cisplatin is considered as the first-line systemic therapy regimen recommended in most treatment guidelines (NCCN, CSCO, and ESMO) (17). However, traditional systemic chemotherapy has limited therapeutic efficacy in treating metastatic brain tumor because of poor penetration of the blood–brain barrier (BBB) (18). At present, the most effective way to treat brain metastasis is surgery, and whole-brain radiation therapy (WBRT) can be considered for those not suitable for surgery (19). Additionally, targeted therapy also showed some value for treating brain metastasis in some kinds of tumor such as osimertinib in epidermal growth factor receptor (EGFR)-mutated non-small cell lung cancer (NSCLC) (20) and lapatinib in patients with HER2-positive breast cancer brain metastases (21, 22).

Immune therapy through ICIs in treating brain metastasis has also shown some promising therapeutic efficacy, examples including brain metastases from melanoma and lung cancer (23, 24). Ipilimumab was the first ICI in treating brain metastasis from melanoma. In an open-label, multicenter, phase II study, the rate of intracranial clinical benefit was 57% with nivolumab plus ipilimumab treatment for melanoma patients with brain metastasis (24). Another phase II study also showed that ipilimumab has therapeutic effects in some patients with advanced melanoma and brain metastases, particularly when metastases are asymptomatic and small (25). Moreover, a monoclonal antibody called pembrolizumab was investigated in a group of patients with asymptomatic brain metastases, and researchers found that 26% of patients had a response both intracranially and extracranially in a year (26). There are also some studies that focused on the treatment efficacy of ICIs in lung cancer. In a phase II trial, 42 patients were treated with pembrolizumab and researchers found that it has activity in brain metastases from NSCLC and can result in prolonged survival in a group of patients (27). In another large study, 155 patients had brain metastasis and 89 of them had NSCLC. Researchers found that patients treated with stereotactic radiosurgery (SRS) and ICIs together had better response rates and response durability compared with those treated with SRS and delayed ICIs (28).

However, one question which has to be considered when treating brain metastasis with ICIs or with most of other anticancer agents is the fact that several barriers in the central nervous system (CNS) can limit the access of monoclonal antibodies (mAbs). The BBB, the blood–tumor barrier (BTB), and the blood–cerebrospinal fluid (CSF) barrier are the three major barriers limiting systemic drug delivery. The BBB can selectively prevent certain substances from entering the brain from blood so that large anticancer agents such as mAbs cannot cross a normal BBB. Although the superior therapeutic efficacy of some targeted agents and ICIs has been reported in some clinical trials, limited information showed the capacity to cross CNS barriers and exert their pharmacodynamic effect. This fact hampers the further investigation about the reasons for the failure or success of these drugs applied in clinical settings (18). Although the brain is believed to be secluded from peripheral immune activity for decades, this perception was revised recently with the findings that higher functions, homeostasis, and repair of the CNS are supported by peripheral innate and adaptive immune cells (29). Actually, the mechanisms related to the priming and recruitment of T cells to the CNS are only partially understood. One would ask whether ICI-treated CD8^+^ T cells can be recruited to the brain metastasis and killed the tumor cells. This is a conceivable scenario because CNS-reactive T cells might be activated in the peripheral immune compartment by non-CNS antigens that from peripheral sites (30).

In this case, our patient with iCCA brain metastasis was treated with camrelizumab 200 mg intravenously every 3 weeks plus lenvatinib (8 mg orally once daily) and this treatment showed favorable efficacy. The rationale for combining lenvatinib with PD1 inhibitors is based on the ability of lenvatinib to inhibit the proneoangiogenic and immunosuppressive effects of tumor microenvironments; such inhibition would improve the clinical benefit of PD-1 antibodies by enhancing the antitumor immune response (31, 32). In a phase Ib study accessing the efficacy and safety of lenvatinib in combination with pembrolizumab in 13 evaluable patients with unresectable HCC (33), no new adverse event was identified, with a partial response (PR) rate of 46% (6/13). Notably, Chen et al. reported the first use of nivolumab plus lenvatinib to successfully treat recurrent, progressive, metastatic cholangiocarcinoma (34). Additionally, pembrolizumab, nivolumab, and three PD-1 antibodies manufactured in China (camrelizumab, sintilimab, and toripalimab) have been approved by the National Medical Products Administration (NMPA) of China. The price of the three PD-1 antibodies manufactured by local pharmaceutical companies is about one-third that of nivolumab or pembrolizumab (less than 2,000 US dollars per month). Clinical trials have accessed the efficacy and safety of camrelizumab in treating primary liver cancer patients (35–37). In this way, camrelizumab might be a good choice for patients who need immunotherapy. However, brain metastasis from iCCA is extremely rare. To the best of our knowledge, the efficacy of PD-1 inhibitors in treating solid central nervous system metastasis from iCCA is seldom reported. In our report, this patient had a symptomatic brain metastasis and the focus was recognized 3 months after liver surgery. After discussion of our MDT, we initiated immunotherapy combined with targeted therapy to treat this patient. Intriguingly, this patient is well-tolerated to this therapy and after 16 cycles’ treatment, the metastatic tumor in the brain disappeared totally. Until the last follow-up in February 2022, no new metastatic lesions and recurrence was found. This case provides a proof of principle that brain metastatic lesions from iCCA are accessible to the action of immunotherapy and that immunotherapy combined with targeted therapy may have a promising effect.

One limitation of this case is that we proceeded with treating the brain lesion as a presumptive metastasis without acquiring a histological verification. The time interval between iCCA diagnosis and brain tumor lesion occurrence was only within 3 months. As a general rule, brain metastases should be suspected in any patient with known systemic cancer in whom neurological findings develop (38). On the basis of the imaging characteristics of PET/CT and brain MRI, the new tumor lesion appearing in this period can be identified as liver cancer brain metastasis, but not specific enough for definitive diagnosis because histopathological analysis of tissue harvested at surgical resection remains the gold standard for diagnosis (38, 39). Compared with the invasive surgical resection strategy, our MDT considered PD1 inhibitor plus targeted drug combination therapy might be the best choice.

Solid central nervous system (CNS) metastasis from iCCA is rare, and together with this case, there are currently only 20 cases having been reported in literature (**Table 1**) (40–48). The ages of these patients range from 46 to 75 (mean 61.1 years), and the male: female ratio is 1:1.9. Among these 20 cases, the median overall survival was 5.7 months, which was much shorter than that of brain metastasis in other malignancies (49). Additionally, the time interval from the diagnosis of iCCA to the discovery of brain metastasis extended up to 3.5 years (median 8 months). Apart from three patients who did not receive any treatment, the therapeutic strategies in these cases were distinct. Generally, 12 of these patients underwent tumor resection of liver and only four of them further accepted craniotomy whereas nearly all of them (11 cases) underwent adjuvant therapy such as WBRT, chemotherapy, targeted therapy, or immunotherapy. For these 12 cases, the median overall survival was 6.2 months, longer than that in all of these 20 cases. Further, the five left cases who just accepted adjuvant therapy such as chemotherapy, WBRT, or laser interstitial thermal therapy (LITT) only achieved moderated therapeutic efficacy. Notably, although among all of these 20 cases, only two patients used the combined immunotherapy and targeted therapy, the survival time of these two patients was both longer than 16 months and the follow-up of them was still continue at the time of reporting. However, more evidence is expected to identify the efficacy of combined immunotherapy and targeted therapy in treating iCCA brain metastasis in future clinical trials.

**Table 1:** Case reports summary of the iCCA patients with brain metastasis (iCCA: intrahepatic cholangiocarcinoma).

	Reference	Sex	Age	Type of primary tumor	Surgery	Adjuvant therapies	Time interval	Number of brain metastasis	Survival times
1	M.S. Gudesblatt et al (40).	F	75	iCCA	No	No	0	1	4 days
2	J. Miyamoto et al (41).	F	67	iCCA	Yes	Yes	2 years	1	> 6 months
3	K. Mimatsu et al (42).	M	60	iCCA	Yes	Yes	10 months	1	> 7 years
4	J. Chindaprasirt et al (43).	M	61	iCCA	No	No	mean 8 months	≥4	12 weeks
5		F	55	iCCA	Yes	Yes	mean 8 months	1	8 weeks
6		F	54	iCCA	No	No	mean 8 months	≥4	11 weeks
7		F	46	iCCA	Yes	Yes	mean 8 months	2	28 weeks
8		F	60	iCCA	Yes	None	mean 8 months	1	13 weeks
9		F	72	iCCA	No	None	mean 8 months	1	8 weeks
10	A.E. Mirrakhimov et al (44).	M	55	iCCA	No	Yes	#	1	1 month
11	G. Frega et al (45).	M	56	iCCA	No	Yes	3.5 years	≥1	4.8 months
12		F	63	iCCA	No	Yes	15.7 months	≥1	0.9 month
13		F	69	iCCA	Yes	Yes	7.3 months	≥1	2.6 months
14	F. Novegno et al (1).	F	65	iCCA	Yes	Yes	3 years	1	1 year
15	K. Fujimoto et al (46).	F	56	iCCA	Yes	Yes	2 years	1	>1 year
16		F	56	iCCA	Yes	Yes	3 years	≥1	4 years
17		M	65	iCCA	No	Yes	0	≥1	1 year
18	S.T. Tan et al (47).	M	71	iCCA	No	Yes	14 months	1	>16 months
19	JT. Zheng et al (48).	F	62	iCCA	Yes	Yes	0	1	>39 months
20	Present case	M	54	iCCA	Yes	Yes	3 months	1	>17 months

Generally, our clinical case describes a patient with a complete regression of the metastatic brain tumor from iCCA after surgical resection of the primary focus and combination therapy with camrelizumab and lenvatinib. In treating brain metastasis from iCCA, we suggest it important to prevent the recurrence of the primary tumor with early employment of immunotherapy-based combination therapy to keep the activation of the surveillance of immune system.

## Data availability statement

The original contributions presented in the study are included in the article/supplementary material. Further inquiries can be directed to the corresponding authors.

## Ethics statement

Written informed consent was obtained from the individual(s) for the publication of any potentially identifiable images or data included in this article.

## Author contributions

All authors listed have made a substantial, direct, and intellectual contribution to the work, and approved it for publication.

## Funding

This work was funded by the National Nature Science Foundation of China (82102959, 82172739, 81871924, 82072666, 81802893, and 81972829), Shanghai Natural Science Foundation (21ZR1481900), Beijing Xisike Clinical Oncology Research Foundation (Y-Roche2019/2-0037), Beijing iGandan Foundation (GDXZ-08-06), and Zhongshan Talent Development Program (2021ZSYQ11).

## Conflict of interest

The authors declare that the research was conducted in the absence of any commercial or financial relationships that could be construed as a potential conflict of interest.

## Publisher’s note

All claims expressed in this article are solely those of the authors and do not necessarily represent those of their affiliated organizations, or those of the publisher, the editors and the reviewers. Any product that may be evaluated in this article, or claim that may be made by its manufacturer, is not guaranteed or endorsed by the publisher.
